# Promising potential of articaine-loaded poly(epsilon-caprolactone) nanocapules for intraoral topical anesthesia

**DOI:** 10.1371/journal.pone.0246760

**Published:** 2021-02-11

**Authors:** Camila Batista da Silva, Maria Cristina Volpato, Bruno Vilela Muniz, Cleiton Pita dos Santos, Luciano Serpe, Luiz Eduardo Nunes Ferreira, Nathalie Ferreira Silva de Melo, Leonardo Fernandes Fraceto, Francisco Carlos Groppo, Michelle Franz-Montan

**Affiliations:** 1 Department of Biosciences, Piracicaba Dental School, University of Campinas—UNICAMP, Piracicaba, São Paulo, Brazil; 2 Health Sciences, University of Mogi das Cruzes–UMC, Mogi das Cruzes, São Paulo, Brazil; 3 Itapeva Faculty of Social and Agrarian Sciences—FAIT, Itapeva, São Paulo, Brazil; 4 Department of Dentistry, State University of Ponta Grossa, Ponta Grossa, Paraná, Brazil; 5 Laboratory of Inflammation and Immunology, Guarulhos University–UNG, Guarulhos, São Paulo, Brazil; 6 Department of Environmental Engineering, São Paulo State University, Sorocaba, SP, Brazil; 7 Department of Immunology and Molecular Biology, São Leopoldo Mandic Research Institute, Campinas, SP, Brazil; Catholic University of Korea, REPUBLIC OF KOREA

## Abstract

To determine whether the permeation capacity and analgesic efficacy of articaine (ATC) could be increased and cytotoxicity decreased by encapsulation in poly(ɛ-caprolactone) nanocapsules (ATC_nano_), aiming at local or topical anesthesia in dentistry. Cellular viability was evaluated (using the MTT test and fluorescence microscopy) after 1 h and 24 h exposure of HaCaT cells to ATC, ATC_nano_, ATC with epinephrine (ATC_epi_), and ATC in nanocapsules with epinephrine (ATC_nanoepi_). The profiles of permeation of 2% ATC and 2% ATC_nano_ across swine esophageal epithelium were determined using Franz-type vertical diffusion cells. Analgesic efficacy was evaluated with a von Frey anesthesiometer in a postoperative pain model in rats, comparing the 2% ATC, 2% ATC_nano_, 2% ATC_epi_, and 2% ATC_nanoepi_ formulations to 4% ATC_epi_ (a commercially available formulation). We show that use of the nanocapsules decreased the toxicity of articaine (*P*<0.0001) and increased its flux (*P* = 0.0007). The 2% ATC_epi_ and 4% ATC_epi_ formulations provided higher analgesia success and duration (*P*<0.05), compared to 2% ATC, 2% ATC_nano_, and 2% ATC_nanoepi_. Articaine-loaded poly(ɛ-caprolactone) nanocapsules constitute a promising formulation for intraoral topical anesthesia (prior to local anesthetic injection), although it is not effective when injected in inflamed tissues for pain control, such as irreversible pulpitis.

## Introduction

Articaine is an amide local anesthetic containing a thiophene ring and an ester radical in its chemical structure [1]. It is widely used in dentistry due to the fast onset and appropriate duration of analgesia, good diffusibility, and short plasma half-life, compared to other amide-type anesthetics [2].

Articaine has been shown to provide a better quality of pulpal anesthesia in inflammatory hypernociception, such as irreversible pulpites [3,4]. In addition, it is effective in lower molar pulpal anesthesia after its buccal infiltration, which is usually achieved using blockade techniques [4–7]. However, there have reports of paresthesia and transitory post-operative sensitivity associated with the use of articaine, which could be related to the concentration in which the compound is used (4%) [8].

In the last decade, there has been a significant increase in the number of studies concerning local anesthetics associated with bioactive molecules and/or sustained release systems, with the aim of reducing drug toxicity and improving bioavailability [9–11]. Among such systems, polymeric nanoparticles have attracted great interest. These consist of vesicles with diameter smaller than 1 μm, which can be classified as nanospheres or nanocapsules, according to their composition and structural organization. Nanospheres are composed of a dense matrix, while nanocapsules consist of a polymeric coat surrounding an oily nucleus. The drug can be dissolved in the nucleus, dispersed in the matrix, or adsorbed on the particle surface [12–14].

Previous results have shown that polymeric nanoparticles are a promising drug release system for local anesthetics. Poly(ε-caprolactone) nanospheres containing lidocaine presented lower toxicity and improved analgesic action, compared to plain lidocaine [15]. Formulations of articaine-loaded poly(ɛ-caprolactone), poly(ethylene glycol)-poly(ɛ-caprolactone), and alginate/chitosan nanocapsules showed high encapsulation efficiencies and lower cytotoxicity, compared to free local anesthetic solution [16,17]. More recently, hydrogels of articaine-loaded poly(ɛ-caprolactone) nanocapsules were found to present increased *in vitro* permeability across a cellulose membrane [18] which could result in better clinical performance, since a high correlation between *in vitro* permeation parameters and *in vivo* intraoral topical anesthesia effect was previously found [19,20]. In addition, drug delivery systems can act as permeation enhancers and increase transdermal delivery [21,22].

Therefore, in this work, evaluation was made of whether encapsulation of articaine in poly(ε-caprolactone) nanocapsules could decrease its cytotoxicity, and increase permeability and analgesic efficacy, focusing on its possible use in local or topical anesthesia in dentistry.

## Materials and methods

### Experimental design

The effect of encapsulation in poly(ε-caprolactone) nanocapsules on the cytotoxicity of articaine was evaluated *in vitro* in viability tests using human keratinocyte (HaCaT) cells, employing the MTT colorimetric assay and fluorescence imaging. The permeation of articaine across swine esophageal epithelium was evaluated using Franz-type vertical diffusion cells. The analgesic efficacy of articaine was determined using a postoperative pain model in rats.

The formulations tested and the respective controls for the cell culture viability tests were as follows: articaine (ATC), articaine with 1:200,000 epinephrine (ATC_epi_), articaine-loaded poly(ε-caprolactone) nanocapsules (ATC_nano_), articaine-loaded poly(ε-caprolactone) nanocapsules with 1:200,000 epinephrine (ATC_nanoepi_), cell culture medium (DMEM), DMEM with epinephrine, DMEM with poly(ε-caprolactone) nanocapsules, and DMEM with poly(ε-caprolactone) nanocapsules and epinephrine. Epinephrine (at a final concentration of 1:200,000) was added to the formulations immediately before the experiment. All the formulations were diluted in DMEM immediately before use.

Articaine at 2% (2% ATC) and 2% articaine-loaded poly(ε-caprolactone) nanocapsules (2% ATC_nano_) were evaluated using the *in vitro* permeability model.

The following formulations were evaluated *in vivo* in terms of analgesic efficacy: 2% articaine-loaded poly(ε-caprolactone) nanocapsules (2% ATC_nano_), 2% articaine-loaded poly(ε-caprolactone) nanocapsules with 1:200,000 epinephrine (2% ATC_nanoepi_), 2% articaine (2% ATC), 2% articaine with 1:200,000 epinephrine (2% ATC_epi_), and 4% articaine with 1:200,000 epinephrine (4% ATC_epi_). The negative controls were poly(ε-caprolactone) nanocapsules, poly(ε-caprolactone) nanocapsules with 1:200,000 epinephrine, 0.9% sodium chloride solution, and 0.9% sodium chloride solution with 1:200,000 epinephrine. The 4% ATC_epi_ formulation was tested because this is the clinically available formulation of articaine associated with epinephrine. The epinephrine bitartrate salt was purchased from Sigma-Aldrich (St. Louis, MO, USA). Epinephrine was added to the formulations immediately before the experiment.

### Preparation of the formulations

Articaine base (in the neutral form) was obtained as described previously [18]. Briefly, articaine hydrochloride (donated by DFL Ind. Com. Ltda., Rio de Janeiro, Brazil) was dissolved in water, the pH was adjusted to pH 8.5, and the aqueous phase was extracted with ethyl acetate. The organic phase was filtered, dried, and evaporated. The oil obtained was crystallized at -18 ^o^C.

Nanocapsule formulations were prepared by the oil-in-water emulsion/solvent evaporation method [23] based on the composition determined earlier [17]. Briefly, two solutions, one containing 400 mg of poly(ε-caprolactone) (PCL, Sigma-Aldrich, St. Louis, MO, USA) and 200 mg of Myritol^®^ 318 (donated by Chemspecs Comercio e Representaçoes Ltda., São Paulo, Brazil) in 20 mL of chloroform (Labsynth, Diadema, São Paulo, Brazil), and another with 200 mg articaine base dissolved in 10 mL of acetone (Labsynth, Diadema, São Paulo, Brazil), were mixed and sonicated for 1 min at 100 W. This pre-emulsion was added to 50 mL of aqueous solution containing 150 mg of polyvinyl alcohol surfactant (PVA, Sigma-Aldrich, St. Louis, MO, USA), with sonication for 8 min to form the emulsion. The organic solvent was removed in a rotary evaporator and the emulsion was concentrated to a final volume of 10 mL, containing 20 mg/mL articaine [15]. The volume was then adjusted to 18.178 mL and 13.516 mg of articaine hydrochloride were added, in order to obtain an encapsulation efficiency of approximately 50%.

This encapsulation was chosen because a short onset was desired for clinical use, which is not achieved with a higher anesthetic encapsulation. This was confirmed in the pilot study, where all the animals presented lip numbness at the first analgesia assessment (5 min after the injection), using around 50% encapsulation of articaine in poly(ε-caprolactone) nanocapsules.

#### Characterization of articaine-loaded poly(ε-caprolactone) nanocapsules

The mean diameter (MD), polydispersion index (PI), and pH of the articaine-loaded poly(ε-caprolactone) nanocapsules were evaluated. The MD and PI were determined by dynamic light scattering, using a ZetaSizer Nano ZS 90 instrument (Malvern Instruments, UK). The measurements (in triplicate) were performed at 25°C, with a fixed angle of 90°, using diluted samples (about 1.0x10^-3^ mol/L) [15–18].

The pH values of the nanocapsules in aqueous suspensions were determined (in triplicate) using a previously calibrated pH meter (Thermo Electron Orion^®^ Model 290A+, Sigma). The stability results for this formulation have been presented previously [18].

The encapsulation efficiency (%EE) of articaine was calculated by subtracting the non-encapsulated (free) local anesthetic from the total local anesthetic present in the initial solution. The amount of non-encapsulated articaine was determined by ultrafiltration (Microcon 10 kDa pore size filter units, Millipore) and centrifugation (14,000 *g* for 25 min), followed by HPLC quantification, as described below (Articaine analysis). All analyses were performed in triplicate.

#### Articaine analysis

Articaine was quantified by high performance liquid chromatography (HPLC), employing a Varian ProStar 325 instrument (Agilent Technologies, Lake Forest, California) equipped with an isocratic PS 210 pump, a UV-Vis detector, and an automatic injector, according to a previously validated method [18]. Galaxy Workstation 410 software (Agilent Technologies, Lake Forest, California) was used for data collection.

Briefly, the chromatographic conditions consisted of a C18 reversed-phase column (5 μm, 110 Å, 150 x 4.6 mm; Phenomenex Gemini), a mobile phase of monobasic sodium phosphate (0.02 mol/L, pH 3.0, adjusted with phosphoric acid) and acetonitrile (88:12, v/v), at a flow rate of 1.5 mL/min, and a controlled temperature of 35°C. The mobile phase was filtered and degassed. The injection volume was 100 μL and the detector wavelength was set at 274 nm. Under these conditions, the limits of detection and quantification were 0.007 and 0.023 μg/mL, respectively (R^2^ = 0.9992).

The HPLC-grade acetonitrile employed in the chromatographic analyses was obtained from JT Baker (Phillipsburg, NJ, USA).

### *In vitro* models

#### Cell viability assays

*Cell culture*. Immortalized human keratinocytes (HaCaT) were chosen due to their good proliferative capacity in culture and their use in previous *in vitro* cytotoxicity studies of local anesthetics and drug delivery systems [24,25]. The HaCaT cells (BCRJ 0100, Rio de Janeiro Cell Bank, Rio de Janeiro, RJ, Brazil) were grown in 75 cm^2^ TPP^®^ bottles (Techno Plastic Products AG, Trasadingen, Switzerland) containing Dulbecco’s Modified Eagle Medium (DMEM, Vitrocell, Campinas, SP, Brazil) supplemented with 10% (v/v) fetal bovine serum (Vitrocell), 10,000 U/mL penicillin (Vitrocell), and 100 μg/mL streptomycin (Vitrocell), at 37°C, in a humid atmosphere with 5% CO_2_ [24,25]_._

*MTT colorimetric assay*. Semi-confluent cells were seeded into 96-well tissue plates, at approximately 1x10^4^ cells/well, followed by incubation for 48 h, at 37°C, in a humid atmosphere with 5% CO_2_. Subsequently, the cells were exposed to ATC, ATC_epi_, ATC_nano_, and ATC_nanoepi_, at concentrations in the range from 0.06% to 1%, for 1 h or 24 h. The half-maximal inhibitory concentration (IC_50_) was determined for all formulations and exposure periods.

Cell viability was evaluated by incubation with 0.3 mg/mL MTT (3-(4,5-dimethylthiazol-2-yl)-2,5-diphenyltetrazolium bromide; Sigma-Aldrich, St. Louis, MO, USA) for 3 h at 37°C. The cell viability values were obtained by conversion of the absorbance readings (A_570 nm_) into percentages of viable cells. The values obtained for DMEM were used as reference controls (100% cell viability).

*Cell viability by fluorescence imaging*. The fluorescence imaging analysis of cell viability was performed as described previously, using the LIVE/DEAD^®^ assay (Invitrogen, Carlsbad, CA, USA) [25]. Briefly, approximately 1x10^5^ cells were inoculated into 24-well plates and were incubated for 48 h, at 37°C, under a humid atmosphere with 5% CO_2_. Subsequently, the cells were exposed to ATC, ATC_epi_, ATC_nano_, and ATC_nanoepi_, at the ATC IC_50_ obtained in the MTT assay. Methanol (70%) was used as the cell death positive control. After exposure to the formulations for 1 h or 24 h, the cells were washed twice with phosphate-buffered saline (PBS), followed by addition of 300 μL of the LIVE/DEAD^®^ solution to each well containing HaCaT cells. The plates were then incubated at room temperature for 30–45 min. Fluorescence images were acquired using an inverted microscope (Axiovert 40 CFL, Carl Zeiss, Germany) coupled to an MEC camera (AxioCam, Carl Zeiss, Germany). Calcein/AM was detected using wavelengths of 450–490 nm (excitation) and 515–565 nm (emission). Cells labeled with EthD-1 were detected using wavelengths of 528–546 nm (excitation) and 590–617 nm (emission) [25].

#### *In vitro* permeation assay

The permeation capacities of the articaine formulations were evaluated using a swine esophageal epithelium model. This model has been extensively used as a permeation barrier model in transbuccal delivery studies, due to its similarity to human oral mucosa in terms of histological organization, permeability, and lipid composition [26]. Swine esophagus was obtained from a local slaughterhouse (Frigorífico Angelelli Ltda, Piracicaba, SP, Brazil) and transported (within 10 min) to the laboratory in isotonic phosphate buffer (PBS, pH 7.4). The ends were discarded and the esophageal mucosa was carefully separated from the outer muscle layer with a scalpel. After immersion of the mucosal tissue in a distilled water bath for 2 min, at 60°C, the epithelium was gently separated from the connective tissue and was kept in isotonic saline [26].

Firstly, 1.0 mL of the buffer solution was added to the donor compartment and the cells were allowed to equilibrate for 60 min in a water bath at 37°C. Following the equilibration period, the epithelium barrier integrity was checked and only undamaged specimens presenting tissue resistivity of at least 3 kΩ/cm^2^ were used [27].

The buffer solution was replaced by 1.0 mL of the test formulation, applied to the epithelial surface under infinite dose conditions, and the glass chamber was closed. The experiments were conducted under *sink* conditions. Aliquots of 300 μL were periodically removed from the receptor chamber (at 15, 30, 45, 60, 90, 120, 150, and 180 min), with the volume being immediately made up using fresh buffer solution. The concentration of articaine was determined by HPLC, as described in Articaine analysis.

The cumulative amount of ATC permeated across the swine esophageal epithelium was plotted as a function of time (n = 9 for each formulation). The steady-state flux (*J*_*ss*_) was obtained from the slope of the linear portion of the curve and the lag time was obtained from the intercept of this straight line at the x-axis.

### *In vivo* model

#### Ethical approval

The analgesic efficacy study in rats was approved on March 26^th^ 2012 by the Ethics Committee on Animal Use of the University of Campinas (CEUA-UNICAMP, protocol #2638–1). The procedures were conducted in accordance with the Ethical Guidelines for Investigations of Experimental Pain in Conscious Animals, issued by the International Association for the Study of Pain [28], and complied with the Principles of Laboratory Animal Care (NIH publication #85–23, revised in 1985).

#### Animals

Fifty-four adult male Wistar SPF rats (250–300 g) were obtained from the Multidisciplinary Center for Biological Investigation of the University of Campinas (CEMIB/UNICAMP, Campinas, SP, Brazil). The animals were submitted to a 12-h day/night cycle, at 22°C, and were allowed free access to water and food during the study.

#### Local analgesia efficacy in a postoperative pain model

This experiment consisted of measuring the paw withdrawal response to application of force, using an electronic von Frey anesthesiometer (Insight Equipment Ltda, Ribeirão Preto, Brazil), after induction of hypernociception (postoperative pain), as described previously [29,30]. Briefly, rats were placed in cages divided into compartments (23 cm width x 20 cm depth x 18 cm height) with wire mesh floors and mirrors underneath, allowing 15–30 min for acclimatization. After this period, the anesthesiometer was used to apply progressively greater force, ranging from 0.0073 to 0.456 N, to the plantar surface of the right hind paw, at intervals of 5 min, in order to establish the baseline response, which was calculated as the mean of three measurements.

Following this procedure, an incision (1 cm long x 3 mm deep) closed by three sutures (6–0 nylon, Brasuture Ind. Imp. Exp. Ltda., São Sebastião da Grama, SP, Brazil) was performed in the plantar surface of the right hind paw of the rats, under isoflurane anesthesia (Isoforine, Cristália Chemicals and Pharmaceuticals Ltd., Itapira, SP, Brazil). After allowing 24 h for recovery, the animals were placed in the same cages for acclimatization followed by lateral application of force to the wound, as described previously. Animals presenting 20% reduction in the force required to elicit paw withdrawal were considered as presenting hyperalgesic pain and were submitted to formulations testing [29,30].

These rats were randomly divided into 9 groups (n = 6) and received 0.1 mL injections of the articaine formulations or the controls, at the side of the incision. Five minutes after the injection, force was applied laterally to the wound every 10 min, using the von Frey anesthesiometer. Local analgesia was considered successful when the animal did not withdraw the paw after application of force of 0.456 N. The duration of analgesia was considered to be the period of time between injection of the formulation and the last time that the animal did not withdraw the paw. The formulations were coded by one researcher and the experiments were performed by other investigators blinded for the formulations injected.

### Statistical analyses

The results were analyzed using the unpaired t-test (characterization), nonlinear fit analysis (MTT assay), Mann-Whitney test (*in vitro* permeation parameters), ANOVA and the Student-Newman-Keuls test (analgesia duration), and the log-rank test (analgesia success). The significance level was set at 5%.

## Results

### Characterization of articaine-loaded poly(ε-caprolactone) nanocapsules

The characteristics of the formulations containing articaine-loaded poly(ε-caprolactone) nanocapsules and empty poly(ε-caprolactone) nanocapsules, determined in the present study, are presented in Table 1. The results related to nano-analysis are published in a previous study of our group [18]. After loading the poly(ε-caprolactone) nanocapsules with articaine, no changes were observed in the nanocapsule diameter or the polydispersion index (*P*>0.05).

**Table 1:** Mean diameter, polydispersion index, encapsulation efficiency, and pH values obtained for the poly(ε-caprolactone) nanocapsules.

Parameter	Articaine-loaded poly(ε-caprolactone) nanocapsules	Poly(ε-caprolactone) nanocapsules
Mean diameter (nm)	450.07 ± 27.98	432.12 ± 10.07
Polydispersion index	0.11 ± 0.04	0.10 ± 0.02
pH	6.93 ± 0.06	5.55 ± 0.01
Encapsulation efficiency (%)	51.33 ± 3.06	----

The mean diameter (MD), polydispersion index (PI), and pH of the articaine-loaded poly(ε-caprolactone) nanocapsules were evaluated. The MD and PI were determined by dynamic light scattering, using a ZetaSizer Nano ZS 90 instrument (Malvern Instruments, UK). The measurements (in triplicate) were performed at 25°C, with a fixed angle of 90°, using diluted samples (about 1.0x10^-3^ mol/L) [15–18].

The pH values of the nanocapsules in aqueous suspensions were determined (in triplicate) using a previously calibrated pH meter (Thermo Electron Orion^®^ Model 290A+, Sigma). The stability results for this formulation have been presented previously [18].

### Cell viability assays

**Fig 1:**
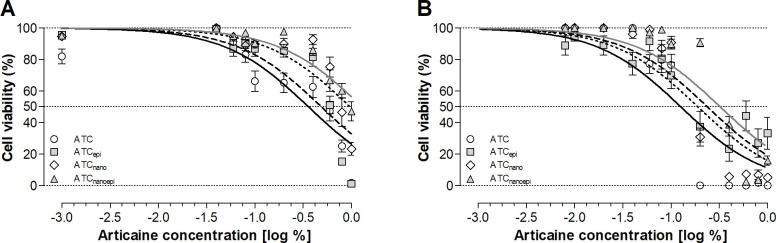
Percentage (%) cell viability (mean ± SEM) after (A) 1-h and (B) 24-h exposure to the formulations containing articaine at different concentrations, using the MTT colorimetric assay ATC: Articaine; ATC_epi_: Articaine with 1:200,000 epinephrine; ATC_nano_: Articaine-loaded poly(ε-caprolactone) nanocapsules; ATC_nanoepi_: Articaine-loaded poly(ε-caprolactone) nanocapsules with 1:200,000 epinephrine. Nonlinear fit analysis (*P*<0.0001).

**Fig 2:**
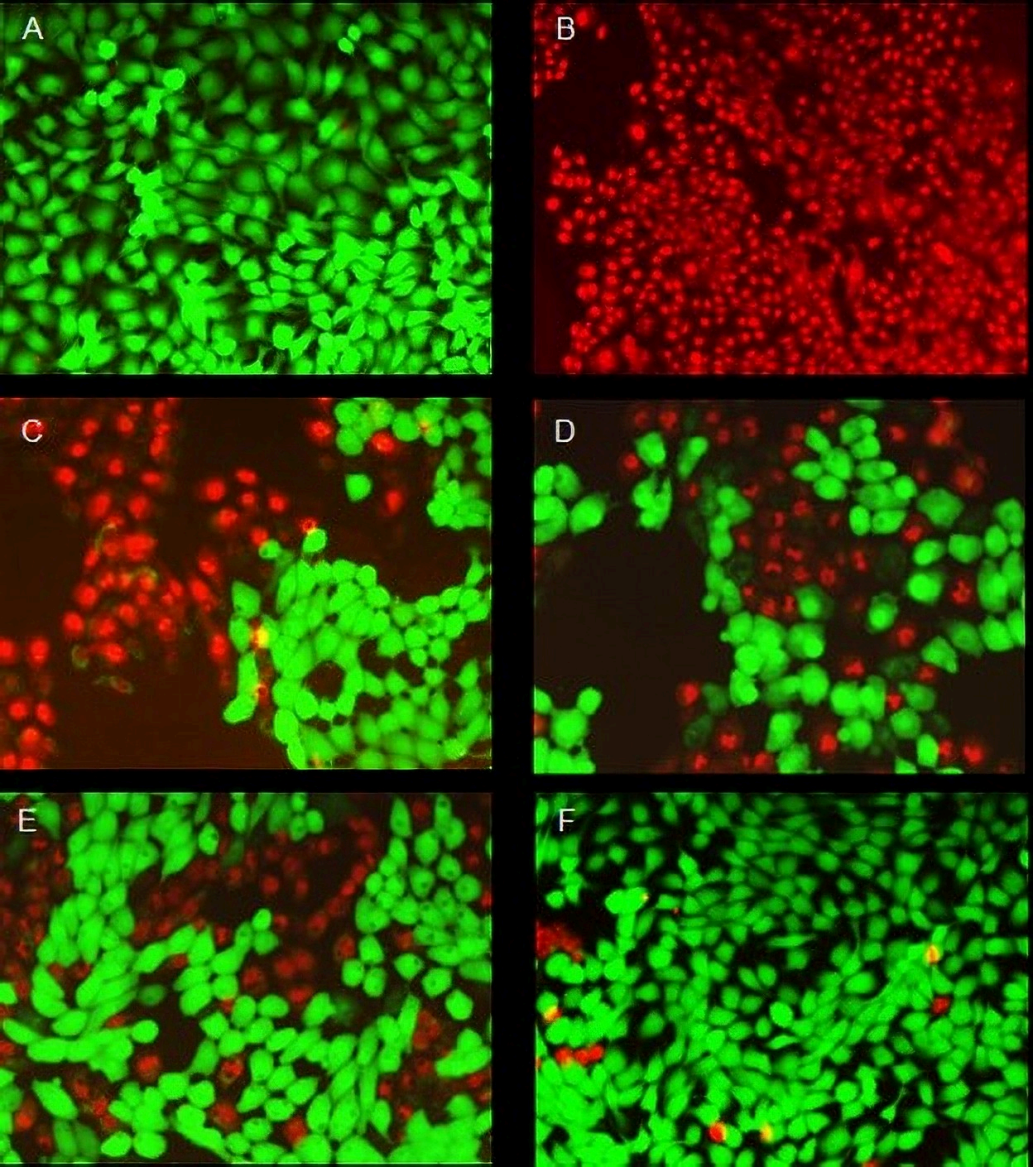
Fluorescence images showing the viability of the HaCaT cells treated with articaine formulations at the IC_50_ concentration for 1 h at 37°C. (A) live cells (negative control); (B) dead cells (positive control treated with 70% methanol); treatments with (C) ATC; (D) ATC_epi_; (E) ATC_nano_; (F) ATC_nanoepi_. Each image is composed of two superimposed images taken through different filters for green (calcein) and red (homodimer-1) fluorescence. All images were acquired at the same magnification (10x).

**Fig 3:**
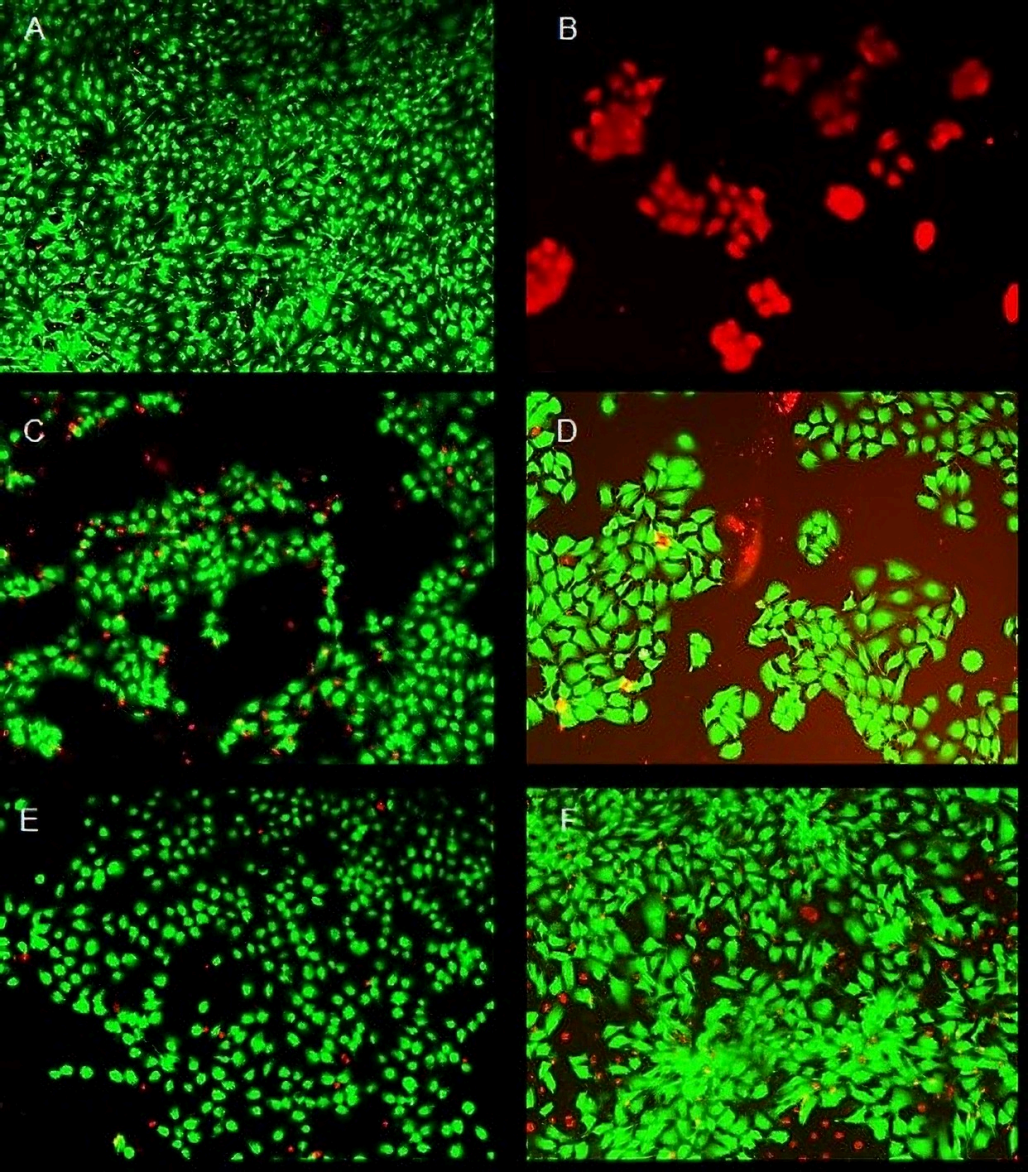
Fluorescence images showing the viability of the HaCaT cells treated with articaine formulations at the IC_50_ concentration for 24 h at 37°C. (A) live cells (negative control); (B) dead cells (positive control treated with 70% methanol); treatments with (C) ATC; (D) ATC_epi_; (E) ATC_nano_; (F) ATC_nanoepi_. Each image is composed of two superimposed images taken through different filters for green (calcein) and red (homodimer-1) fluorescence. All images were acquired at the same magnification (10x).

The ATC formulation was the most cytotoxic to HaCaT cells after 1 and 24 h, while ATC_nanoepi_ was the least cytotoxic. The addition of epinephrine or encapsulation in the nanocapsules decreased the articaine cytotoxicity, which increased in the order ATC_nanoepi_ < ATC_nano_ < ATC_epi_ < ATC (Fig 1, Table 2), Nonlinear regression analysis showed a significant difference between all the formulations (Table 2), as illustrated in Figs 2 and 3.

**Table 2:** Half-maximal inhibitory concentration (IC_50_: Concentration for inhibition of 50% of the cells) and confidence interval (%) after 1-h and 24-h exposure of HaCaT cells to articaine formulations.

Formulations	IC_50_ (%)		IC_50_ confidence interval (%)	
	1-h	24-h	1-h	24-h
ATC	0.3597	0.1176	0.3034 to 0.4265	0.1082 to 0.1277
ATC_epi_	0.5786	0.2467	0.5469 to 0.6122	0.1770 to 0.3439
ATC_nano_	0.7692	0.1639	0.7272 to 0.8137	0.1490 to 0.1803
ATC_nanoepi_	0.9582	0.3487	0.8538 to 1.075	0.3220 to 0.3776

*P*<0.0001 for all formulations. Different exposure times were analyzed separately.

The cell viabilities obtained after 1 h exposure to the control formulations were 98.5% (DMEM with epinephrine), 97.3% (DMEM with poly(ε-caprolactone) nanocapsules), and 97.3% (DMEM with poly(ε-caprolactone) nanocapsules and epinephrine). After 24 h exposure to the control formulations, the cell viabilities were 95.6% (DMEM with epinephrine), 97.8% (DMEM with poly(ε-caprolactone) nanocapsules), and 93.0% (DMEM with poly(ε-caprolactone) nanocapsules and epinephrine).

### *In vitro* permeation assay

**Fig 4:**
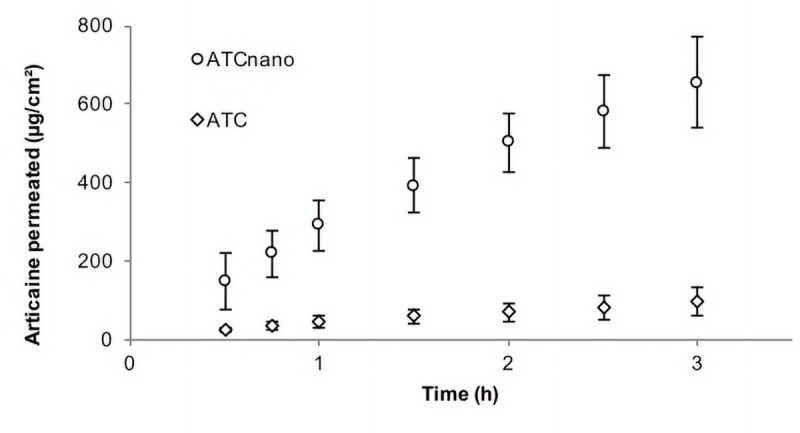
Permeation profiles (mean ± SD, n = 6–8) of ATC permeated after 3 h, for ATC permeation across fresh swine esophageal epithelium from formulations applied under infinite dose conditions. White circles: ATC_nano_ (articaine-loaded poly(ε-caprolactone) nanocapsules; white diamonds: ATC (articaine).

The ATC_nano_ formulation presented higher permeation, compared to the ATC formulation (Fig 4, Table 3). There was no lag time (0 h) for either formulation since permeation occurred immediately after experiment started, as the intercept of the straight line at the x-axis presented negative values for either formulations (ATC = -0,79±0,40 and ATC_nano_ = -0,49±0,33).

**Table 3:** Calculated steady-state fluxes (*J*_*ss*_) and cumulative amounts of ATC permeated after 3 h (Q_3h_), for ATC permeation across fresh swine esophageal epithelium from formulations applied under infinite dose conditions.

Formulation	*J*_*ss*_ (μg.cm^-2^.h^-1^)	ER*	Q_3h_ (μg)	R^2^ (0.5 – 3h)**
ATC_nano_	191.96(148.46–254.55)**	8.06	1075.7(950.46–1595.3)***	0,984±0,008
ATC	23.82(14.47–31.31)	------	160.78(98.49–335.58)	0,987±0,008

Mann-Whitney test: data presented as median (minimum-maximum) n = 6–8

**P<0.001

***P<0.0001. Each permeation parameter was analyzed separately.

*ER: Enhancement ratio, comparing the *J*_*ss*_ values for ATC_nano_ and ATC. **R^2^: mean(±SD) of regression coefficients calculated between 0.5 and 3 h.

### *In vivo* assays of local analgesia in a postoperative pain model

**Fig 5:**
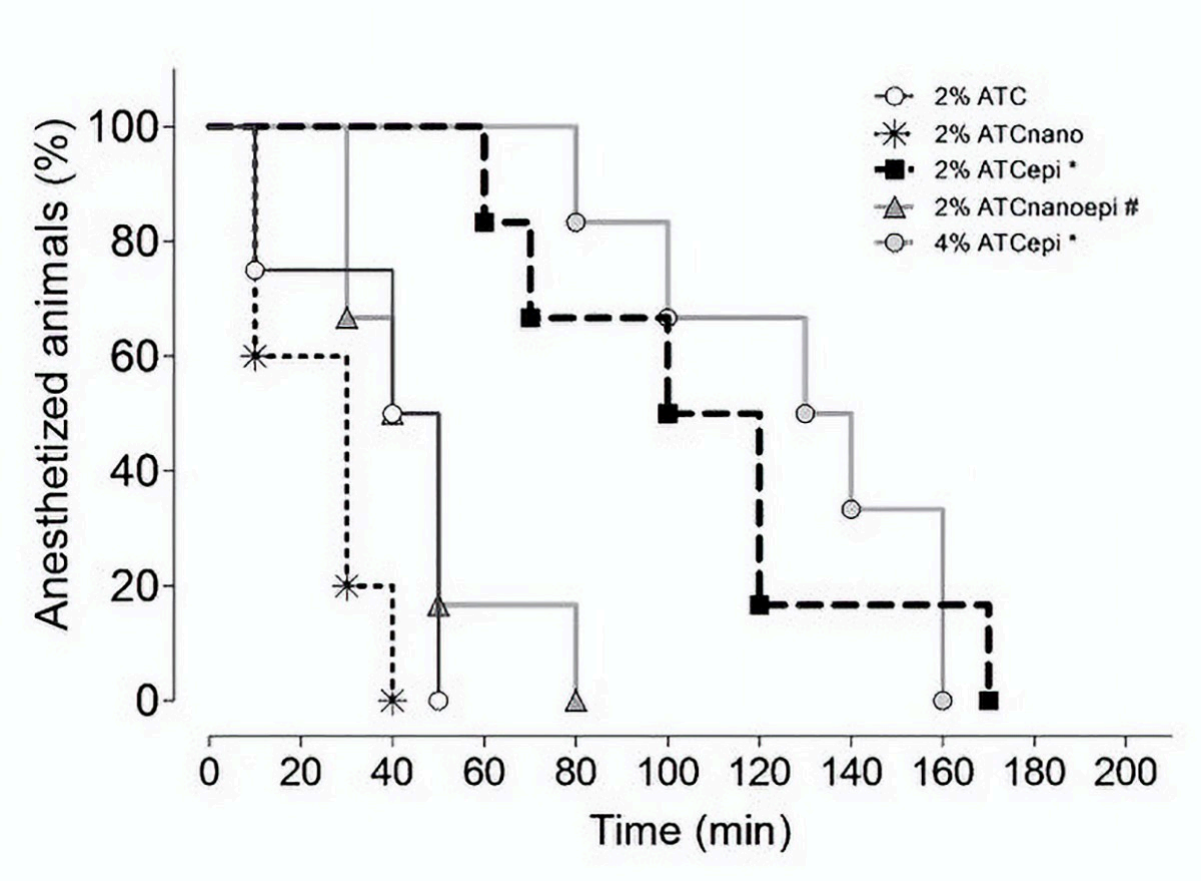
Analgesia success (%) in inflamed tissue after subcutaneous injection. 2% articaine (2% ATC), 2% articaine-loaded poly(ε-caprolactone) nanocapsules (2% ATC_nano_), 2% articaine with 1:200,000 epinephrine (2% ATC_epi_), 2% articaine-loaded poly(ε-caprolactone) nanocapsules with 1:200,000 epinephrine (2% ATC_nanoepi_), and 4% articaine with 1:200,000 epinephrine (4% ATC_epi_) (n = 6 rats/group). Log-rank test: *P<0.01 compared to 2% ATC, 2% ATC_nano_, and 2% ATC_nanoepi_; ^#^*P* = 0.0344 compared to 2% ATC_nano_.

**Fig 6:**
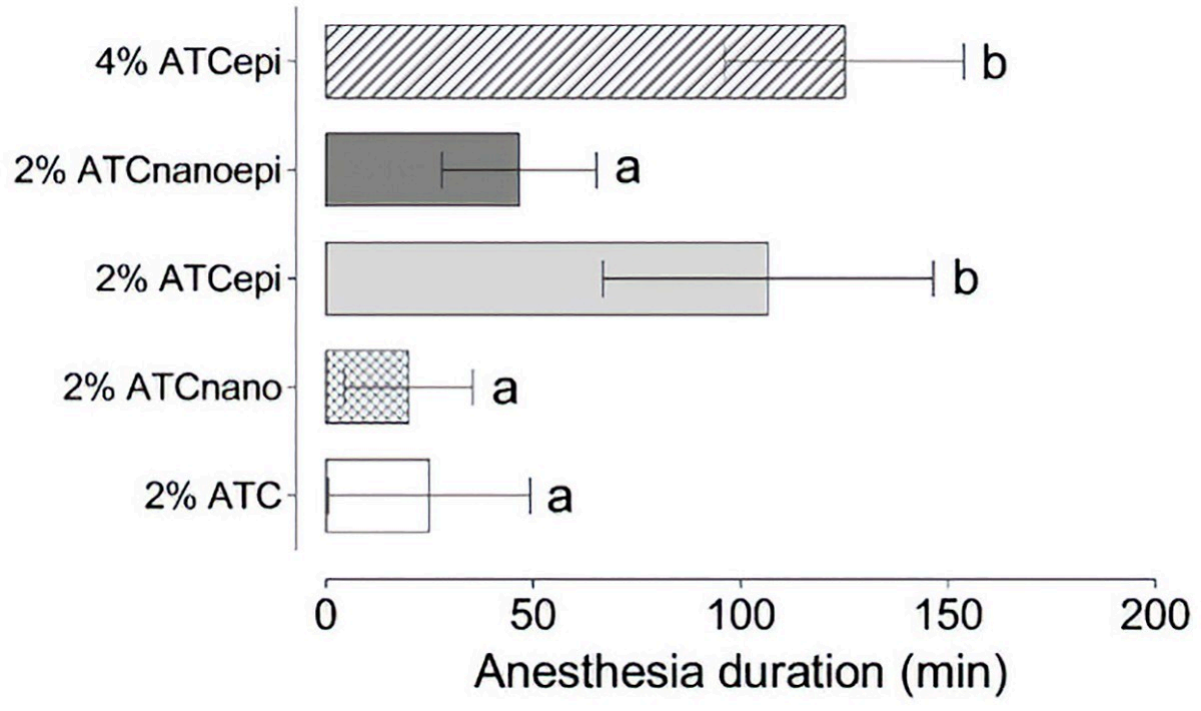
Analgesia duration (mean±SD, in minutes) after subcutaneous injection. 2% articaine (2% ATC), 2% articaine-loaded poly(ε-caprolactone) nanocapsules (2% ATC_nano_), 2% articaine with 1:200,000 epinephrine (2% ATC_epi_), 2% articaine-loaded poly(ε-caprolactone) nanocapsules with 1:200,000 epinephrine (2% ATC_nanoepi_), and 4% articaine with 1:200,000 epinephrine (4% ATC_epi_) (n = 6 rats/group). ANOVA and LSD t-test: different letters indicate *P*<0.05.

All the animals presented a 20% reduction in the withdrawal response to force application after incision/suture and were included in the experiment. The control formulations did not induce local analgesia. The 2% ATC_epi_ and 4% ATC_epi_ formulations presented similar analgesia success (Fig 5) and duration (Fig 6) (*P* = 0.59), and both provided higher analgesia success and duration than 2% ATC, 2% ATC_nano_, and 2% ATC_nanoepi_, while 2% ATC_nanoepi_ provided higher analgesia success than 2% ATC_nano_ (*P* = 0.034).

## Discussion

Articaine presents interesting properties and consequently is widely studied in the world. The encapsulation of articaine in poly(ε-caprolactone) nanocapsules is a promising strategy for dental practice since it provides decreased cytotoxicity, which can reduce the risk of nerve cell damage and paresthesia [2,8], together with increased permeability resulting in higher superficial analgesia after topical application at oral mucosa [19,20].

The mean diameter and polydispersion index of the nanocapsules did not change after the incorporation of articaine. The polydispersion index remained at low values, showing good homogeneity of the system, which confirmed the results previously reported by our group [15–18]. The mean diameter obtained for the articaine-loaded poly(ε-caprolactone) nanocapsules (450.07 ± 27.98) was in the range observed previously for articaine encapsulated in poly(ethylene glycol)-poly(ε-caprolactone) nanospheres (569.2 ± 30.5 nm), articaine in alginate/chitosan nanospheres (342.4 ± 20.5 nm), articaine in poly(ε-caprolactone) nanocapsules (445.5 ± 2.1 nm), and lidocaine in poly(ε-caprolactone) nanospheres (449.6 ± 0.5 nm) [15–18] consistent with values observed for colloidal suspensions [15].

Accordingly, the polydispersion index (0.11 ± 0.04) was in the range observed for articaine encapsulated in poly(ethylene glycol)-poly(ε-caprolactone) nanospheres (0.144 ± 0.025), articaine in alginate/chitosan nanospheres (0.236 ± 0.030), articaine in poly(ε-caprolactone) nanocapsules (0.068 ± 0.005), and lidocaine in poly(ε-caprolactone) nanospheres (0.072) [15–18] and was indicative of homogeneity of the nanoparticles.

Local anesthetics have been tested in different kind of cells, as and fibroblasts, neuroblastoma, mesenchymal stem, keratinocytes [18,31–33]. The immortalized human keratinocytes (HaCaT) have a good proliferative capacity in culture, being used in *in vitro* studies of cytotoxicity [24,33,34] and other cellular responses to stimulus [35,36].

The MTT assay results showed that encapsulation reduced the toxicity of articaine to HaCaT cells exposed for 1 h and 24 h, which was confirmed by the results of the LIVE/DEAD^®^ assay. The fact that the cytotoxicity of articaine was greatly reduced by its encapsulation is an important finding, since this local anesthetic has been associated with cell injury and paresthesia after local injections in dental procedures [2,8].

The ability of polymeric nanoparticles to reduce the cytotoxicity of local anesthetics has been reported previously. After exposure to plain articaine, 3T3 fibroblast cells showed a 50% reduction in viability, while the exposure of these cells to articaine-loaded poly(ethylene glycol)-poly(ε-caprolactone) nanocapsules and to articaine-loaded alginate/chitosan nanospheres resulted in viability losses of 20% and 30%, respectively [16]. It was also found that the incorporation of bupivacaine into alginate/chitosan nanoparticles reduced its toxicity towards 3T3 fibroblasts [37]. This reduced cytotoxicity might be related to the percentage of encapsulated local anesthetic, which resulted in a reduced amount of free local anesthetic available to interact with the cells.

Furthermore, the combination of epinephrine and articaine-loaded poly(ε-caprolactone) nanocapsules resulted in a further reduction in cytotoxicity. This effect was observed for all the formulations containing this vasoconstrictor, for both exposure periods. The protective effect of epinephrine was previously demonstrated in human articular chondrocytes exposed to 1% lidocaine with epinephrine, in comparison to 1% lidocaine, with the protective effect of epinephrine being attributed to its interaction with the local anesthetic [38]. In the present study, epinephrine may also have interacted with the nanocapsules, reducing the release of articaine. However, the kinetics of articaine release from the nanocapsules was not tested in the presence of epinephrine.

All the anesthetic formulations tested in the present study showed increased cytotoxicity (lower IC_50_) with longer treatment periods. The same behavior was observed elsewhere for Schwann cells after 4, 24, 48, and 72 h exposure to lidocaine, mepivacaine, chloroprocaine, ropivacaine, and bupivacaine [39] as well as for human articular chondrocytes exposed to lidocaine for 15, 30, and 60 min [38].

Articaine presents useful clinical properties including high diffusibility, lower plasma half-life than other anesthetics of the amide group, and appropriate interaction with lipid membranes [2,40] The high diffusibility of articaine is demonstrated by the fact that this local anesthetic is able to induce pulpal anesthesia in posterior mandibular teeth by buccal infiltration, which is currently achieved with nerve block for other anesthetic solutions [4–7].

However, increased tissue diffusibility does not necessarily reflect increased ability to cross the epithelium barrier after a topical application. In the present study, the permeation degree of articaine was significantly lower than that of the encapsulated local anesthetic, which revealed its poor ability to penetrate the barrier when applied topically. Drug delivery systems such as liposomes, nanoparticles, and nanocapsules are considered promising drug delivery carriers in topical application on the skin [22]. Several studies have demonstrated that these nanocarriers are able to modify drug permeation profiles, due to their permeation-enhancing effects [21]. This was confirmed by the present results showing that articaine encapsulation improved the permeation profile, with an approximately 8-fold increase in the articaine flux across the mucosal barrier. Similarly, it has also been reported that encapsulation in liposomes increased the permeation of lidocaine and benzocaine through swine palatal and esophageal epithelia, compared to commercially available formulations containing the free local anesthetics [19,20].

It has been suggested that *in vitro* permeation is a valuable tool for prediction of analgesic efficacy during preclinical tests, since an *in vitro/in vivo* correlation was observed [19,20] Although topical articaine is not commonly used in dentistry, the increased permeation achieved by encapsulation in poly(ε-caprolactone) nanocapsules suggests the possibility of its future application in intraoral topical anesthesia, so *in vivo* studies should be undertaken to confirm this hypothesis.

In addition to its higher diffusibility, articaine has been demonstrated to provide greater pulpal anesthesia success in teeth with symptomatic pulpitis (hyperalgesic teeth) [3,4]. However, the encapsulation of articaine in polymeric nanoparticles did not improve efficacy following injection in an inflamed tissue model. The present results showed that hyperalgesia due to the surgical wound did not affect the onset of analgesia after infiltration of the articaine formulations, since 100% of the animals showed local analgesia (absence of paw withdrawal) in the first assessment period (5 min), indicating that a sufficient amount of articaine was available to block the free nerve endings.

However, the duration of analgesia was significantly shorter, compared to that achieved with articaine at 2 or 4% associated with epinephrine. This could be attributed to the vasodilator action of articaine, as shown by other local anesthetics, such as lidocaine. As a consequence, most local anesthetics are not available as plain solutions for clinical use in dentistry. Inflamed tissues present increased vasodilation and vascular permeability, so effective local vasoconstriction is required in order to allow sustained analgesia in these tissues. Epinephrine at 1:200,000 or higher concentrations provides adequate vasoconstriction and improves the duration of dental anesthesia [41].

Based on the present findings, it was not clear why the formulation 2% ATC_nanoepi_ presented lower analgesic effect when compared to 2% ATC_epi_, since the amount of vasoconstrictor was the same in both formulations. We hypothesize that an interaction between epinephrine and the nanocapsules could have occurred, which decreased the amount of epinephrine available to act on the vessels. Therefore, a lower degree of vasoconstriction in inflamed tissue would result in low analgesia success, comparable to that observed for the anesthetic without additives. Such interaction between nanoparticles and epinephrine should be confirmed in future studies.

The amount of articaine did not seem to influence its efficacy in inducing analgesia, since 2% and 4% articaine with epinephrine presented similar duration and success. Previous work also found that 2% and 4% articaine associated with 1:200,000 epinephrine provided similar efficacy for use in non-inflamed tooth extractions in humans [42]. In third molar surgery, efficacy and safety also were tested and articaine at 2 or 4% associated with epinephrine, administered in equal volumes, showed no significant differences [43].

These findings suggest that a formulation with a 50% lower concentration could be clinically used as an alternative to the commercially available 4% articaine, even in inflamed tissue, combining a similar efficacy with a reduction in local toxicity, considering the toxicity to be concentration-related [8]. The systemic toxicity may also be reduced with 2% articaine, compared to 4% articaine (both associated with 1:200,000 epinephrine), as observed in children [44].

## Conclusions

Poly(ε-caprolactone) nanocapsules constitute a promising release system for articaine in intraoral topical anesthesia, providing decreased cytotoxicity and increased permeation across a mucosal epithelium model. However, the system was not effective in a postoperative pain model.

The association of polymeric nanocapsules with epinephrine provided a further decrease in cytotoxicity. However, neither ATC_nano_ nor ATC_nanoepi_ were able to improve local analgesia in inflamed tissue. Epinephrine improved analgesic efficacy at both articaine concentrations (2% and 4%), so evaluation of the lower concentration of use in inflamed tissues might be of utility.
